# Pulmonary metastasectomy in colorectal cancer: the importance of interpreting neoadjuvant strategies in the era of modern systemic therapy

**DOI:** 10.1007/s00384-026-05158-y

**Published:** 2026-05-26

**Authors:** Yusuf Ilhan, Halil Goksel Guzel

**Affiliations:** Department of Medical Oncology, Antalya City Hospital, 5379. Street, 07080 Kepez, Antalya, Turkey

Dear Editor,

We read with great interest the article entitled ‘Neoadjuvant therapy for resectable colorectal cancer pulmonary oligometastases: a retrospective cohort study’ by Cai et al. [1]. We would like to sincerely congratulate the authors for this well-designed study addressing oligometastatic disease in colorectal cancer, in which the pivotal and undeniable role of surgery in achieving cure is appropriately emphasized. The evaluation of pulmonary metastasectomy within this context represents a valuable contribution to the existing literature. However, from the perspective of medical oncology, we believe that several important considerations and suggestions merit discussion, which may further enhance the clinical relevance and interpretability of the presented findings.

Firstly, although the study encompasses a long study period between 2010 and 2022 and reports the use of at least two cycles of 5-fluorouracil-based neoadjuvant chemotherapy (NAC), and despite the authors’ acknowledgment of this limitation, detailed information regarding the specific treatment regimens is unfortunately lacking. In contemporary management of metastatic colorectal cancer (mCRC), systemic therapy extends beyond conventional cytotoxic chemotherapy. Targeted agents, including anti-epidermal growth factor receptor therapies and anti-vascular endothelial growth factor agents, as well as immune checkpoint inhibitors (ICIs)—particularly in patients with microsatellite instability–high (MSI-H) tumors—constitute integral components of modern treatment strategies and have a substantial impact on clinical outcomes. With respect to microsatellite instability status, the proportion of MSI-H patients was reported to be 2.2% in the non-neoadjuvant chemotherapy (non-NAC) group, while the rate of patients with unknown MSI status was remarkably high in both the non-NAC and NAC groups (39.7% and 25.7%, respectively). This limited and incomplete characterization of MSI status represents a significant limitation, particularly in the current immunotherapy era. In patients with MSI-H mCRCs, ICIs have demonstrated substantial and durable survival benefits. For instance, in a landmark study evaluating nivolumab in combination with ipilimumab, the 2-year progression-free survival rate was reported to be approximately 72%, even in cohorts not undergoing surgical intervention [2]. In this context, the high proportion of patients with unknown MSI status and the absence of ICIs administration in the present study warrant a cautious interpretation of the results. Moreover, in light of these advances in the metastatic setting, there has been a growing interest in exploring ICIs as a neoadjuvant strategy for MSI-H colorectal cancers [3]. Incorporation of comprehensive molecular profiling, particularly MSI status, may not only enhance the interpretability of such analyses but could also potentially reveal even more favorable outcomes when modern systemic therapies are integrated with surgical management.

Secondly, it is noteworthy that a substantial proportion of patients in the study cohort (61.7%) declined adjuvant therapy following pulmonary metastasectomy. Furthermore, as adjuvant chemotherapy was administered to only 38.3% of the overall cohort and was significantly more common among patients who had received prior NAC (48.6% vs. 34.1%; *p* = 0.030), this raises the possibility that a substantial proportion of patients in the non-NAC group may have received limited or no systemic therapy in the perioperative setting, which could have influenced the observed survival differences. Moreover, adjuvant chemotherapy has been shown to confer a clear survival benefit with a high level of evidence in stage III colorectal cancer, as well as in selected high-risk stage II patients, and is widely accepted as a standard component of curative-intent treatment [4]. In line with this, adjuvant systemic therapy following metastasectomy has also been recommended in clinical practice and supported by several studies, given its potential to reduce recurrence risk and improve long-term outcomes [5]. Therefore, differences in the utilization of adjuvant treatment between study groups should be carefully considered when interpreting survival results in this setting. This high rate of refusal represents an important source of potential bias and further underscores the need for cautious interpretation of the reported outcomes.

Although propensity score matching (PSM) was employed to mitigate selection bias, it should be acknowledged that clinically relevant baseline differences between the NAC and non-NAC groups may still persist, particularly with respect to unmeasured or incompletely captured clinical and biological variables (such as primary tumor location [right colon, left colon, or rectum] with their well-established biological heterogeneity; the high proportion of patients with unknown MSI status; the presence of solitary versus multiple pulmonary metastases; and the largely unreported KRAS, NRAS, and BRAF mutation status). As with all retrospective analyses, PSM can only balance covariates that are known and included in the model, and residual confounding cannot be fully excluded. Consequently, the observed survival differences may not solely reflect the effect of neoadjuvant therapy, but may also be influenced by underlying biological and clinical differences related to patient selection.

In conclusion, the authors should again be commended for highlighting the critical role of pulmonary metastasectomy in colorectal cancer, a disease in which surgical resection remains one of the clearest contributors to long-term survival and potential cure in selected patients. We believe that the true value of neoadjuvant strategies in this setting can be more accurately appreciated when interpreted within the framework of contemporary systemic treatments, particularly with the integration of molecular profiling and immunotherapy in appropriate patient subsets. A multidisciplinary approach that combines optimal surgical management with modern, biology-driven systemic therapies may further enhance outcomes and help refine patient selection for curative-intent metastasectomy in the evolving treatment landscape of metastatic colorectal cancer.

## Data Availability

No datasets were generated or analysed during the current study.
